# Supplementary material for: Mechanisms governing the pioneering and redistribution capabilities of the non-classical pioneer PU.1
Source: Nat Commun. 2020 Jan 21;11:402. doi: 10.1038/s41467-019-13960-2 (PMC6972792; doi:10.1038/s41467-019-13960-2)
Supplement: Supplementary file 7 — Source data [file 41467_2019_13960_MOESM7_ESM.zip › Source_Data/FigureS2/FigureS2B_MotifScanOutput/KmeansCluster1/homerResults/motif6.similar.html]

motif6

## Information for motif6

A
G
T
C
C
G
A
T
A
G
C
T
T
G
A
C
G
A
C
T
A
G
C
T
T
G
A
C
A
G
C
T
A
G
C
T
G
A
T
C
A
G
C
T
A
G
C
T
  
Reverse Opposite:  

T
C
G
A
T
C
G
A
C
T
A
G
T
C
G
A
T
C
G
A
A
C
T
G
T
C
G
A
C
T
G
A
A
C
T
G
T
C
G
A
G
C
T
A
T
C
A
G
  

|  |  |
| --- | --- |
| p-value: | 1e-275 |
| log p-value: | -6.353e+02 |
| Information Content per bp: | 1.623 |
| Number of Target Sequences with motif | 1198.0 |
| Percentage of Target Sequences with motif | 23.53% |
| Number of Background Sequences with motif | 2578.9 |
| Percentage of Background Sequences with motif | 6.50% |
| Average Position of motif in Targets | 98.8 +/- 53.9bp |
| Average Position of motif in Background | 104.8 +/- 98.0bp |
| Strand Bias (log2 ratio + to - strand density) | -0.0 |
| Multiplicity (# of sites on avg that occur together) | 1.35 |
| Motif File: | file (matrix) reverse opposite |

### Similar de novo motifs found

|  |  |  |  |  |  |  |  |
| --- | --- | --- | --- | --- | --- | --- | --- |
| Rank | Match Score | Redundant Motif | P-value | log P-value | % of Targets | % of Background | Motif file |
| 1 | 0.754 | A G T C A G C T A G T C A G T C A G C T A G T C A G T C A G T C A G T C A G T C A G T C A G C T A G T C | 1e-111 | -256.988796 | 11.94% | 3.77% | motif file (matrix) |
| 2 | 0.760 | G A C T A G T C G A T C G A C T A G T C G A T C G A C T A G T C G A T C G A C T A G T C G A T C G A C T | 1e-87 | -202.077408 | 4.99% | 0.82% | motif file (matrix) |
| 3 | 0.944 | G T A C A C G T A G C T A T G C C G A T A G C T A G T C A C G T A G C T A T G C A G C T | 1e-59 | -136.517640 | 5.56% | 1.55% | motif file (matrix) |
| 4 | 0.807 | C G T A A C T G A T C G C G T A A C T G C G T A C G T A A C T G | 1e-54 | -125.571835 | 11.18% | 5.17% | motif file (matrix) |
| 5 | 0.801 | A C G T A C G T T A G C A C G T A C G T T A G C C A G T C G A T A G T C G A C T A C G T A T G C A C G T A C G T | 1e-44 | -103.458456 | 3.10% | 0.65% | motif file (matrix) |
| 6 | 0.758 | A G T C A G C T C A T G G T A C G A C T A C G T A G T C A C G T T A G C G T A C G A C T A C G T A G T C A C G T | 1e-27 | -64.011592 | 1.89% | 0.39% | motif file (matrix) |
